# Plasma VWF: Ag levels predict long-term clinical outcomes in patients with acute myocardial infarction

**DOI:** 10.3389/fcvm.2022.1013815

**Published:** 2023-01-04

**Authors:** Zulipiyemu Xier, Yu-Xia Zhu, Shou-Wei Tang, Can Kong, Dilihumaer Aili, Guzailinuer Huojia, Hui Peng

**Affiliations:** Department of Cardiology, People's Hospital of Xinjiang Uygur Autonomous Region, Urumqi, Xinjiang, China

**Keywords:** acute myocardial infarction, risk factors, thrombus, prognosis, VWF: Ag

## Abstract

**Background:**

A vital role in coronary artery disease is played by Von Willebrand factor (VWF), which serves as a bridge between platelets and the subendothelial matrix after vessel damage. The purpose of the study was to assess the validity of plasma VWF antigen (VWF: Ag) levels as a predictor of clinical outcomes after acute myocardial infarction (AMI).

**Methods:**

Three hundred and seventy-four patients were studied following coronary angiography, including 209 patients suffering from acute myocardial infarction and 165 healthy participants. Coronary angiography was followed by measurement of plasma VWF: Ag levels. Over a 2-year follow-up period, major adverse cardiopulmonary and cerebrovascular events (MACEs) were the primary endpoint. All-cause mortality was investigated as a secondary endpoint.

**Results:**

When compared to controls, patients with AMI had mean plasma VWF: Ag levels that were ~1.63 times higher (0.860 ± 0.309 vs. 0.529 ± 0.258 IU/ml; *P* < 0.001). The plasma VWF: Ag levels were substantially higher in patients who experienced MACEs after myocardial infarction vs. those without MACEs (1.088 ± 0.253 vs. 0.731 ± 0.252 IU/ml; *P* < 0.001). For predicting long-term MACEs using the optimal cut-off value (0.7884 IU/ml) of VWF: Ag, ROC curve area for VWF: Ag was 0.847, with a sensitivity of 87.2% and a specificity of 66.3% (95%CI: 0.792–0.902; *P* = 0.001). Two-year follow-up revealed a strong link between higher plasma VWF: Ag levels and long-term MACEs. At the 2-year follow-up, multivariate regression analysis revealed an independent relationship between plasma VWF: Ag levels and MACEs (HR = 6.004, 95%CI: 2.987–12.070).

**Conclusion:**

We found evidence that plasma VWF: Ag levels were independent risk factors for AMI. Meanwhile, higher plasma VWF: Ag levels are associated with long-term MACEs in people with AMI.

## Introduction

Although there are new therapeutic approaches available for coronary artery disease (CAD), it remains one of the most common life-threatening cardiovascular disorder. The prevalence and mortality of CAD in China are continuously rising (1). Acute myocardial infarction (AMI) is the leading cause of cardiac mortality worldwide. The chances of survival depend on the severity and clinical manifestations, as well as the treatment plan. Atherosclerosis is a chronic inflammatory condition of the blood vessels characterized by plaque deposition in their interior layers (2, 3). The cascade of events that lead to arterial thrombosis involves multiple mechanisms, including platelets, endothelium, subendothelial matrix, and other hemostatic factors (fibrinogen and Von Willebrand factor) (4).

Among the major components of blood plasma, endothelial cells, megakaryocytes, and platelets is Von Willebrand factor (VWF), a large multimeric glycoprotein. Haemostasis is mediated by VWF, which facilitates platelet adhesion to sites of vascular injury. Coagulation factor VIII (FVIII) is also bound to it and protected from degradation by it (5). VWF stored in the Weibel–Palade bodies of endothelial cells or in the α-granules of megakaryocytes is rich in VWF multimers that are extremely large, and are called ULVWF multimers, whereas the constitutively secreted VWF multimers are shorter, but still of high molecular weight (6). The ULVWF multimers do not typically circulate in the plasma. When endothelial cells are damaged, it is constantly secreted from the Weibel-Palade bodies of endothelial cells into the bloodstream, then rapidly proteolysis that reduces them into smaller multimers soon after their secretion (7, 8).

The role of VWF in the progression of atherosclerosis has been intensively studied (9). There has been evidence that VWF is crucial to the aggregation of platelets at sites of high shear and coronary lesions (10). VWF appears to be a strong indicator of cardiovascular risk, and patients with acute coronary syndrome (ACS) have elevated VWF levels in their plasma (11). Observations of elevated VWF: Ag levels in patients with AMI was linked to plaque rupture (12).

In addition to playing a critical role in AMI and ischaemia/reperfusion injury, VWF has also been implicated in preclinical and clinical research. Modeling AMI in murine models, following an AMI, VWF: Ag levels rise transiently in the cardiac and peripheral blood (13). VWF levels show a typical time course during an acute cardiovascular event. In the setting of ST-elevation MI (STEMI), plasma VWF levels become elevated at 24 h and peak at 48–72 h before returning to baseline at approximately Day 14 (14).

In the case of post-myocardial infarction (MI), the identification of new biomarkers may significantly improve the risk stratification, subsequent care of the patient and long-term outcomes. The results of a large cohort study involving 811 patients with carotid stenosis found that an independent 2.1-fold increase in MACE was associated with VWF levels in the highest quartile (15). A large sample meta-analysis showed that an independent prognostic factor for MACE could be the plasma levels of VWF in patients with coronary artery disease when measured 24 and 48h after admission (16). These findings imply that VWF might have the potential to be a promising biomarker to predict the outcomes of AMI patients. Further clinical research is warranted to establish VWF as a predictor for the prognosis of AMI patients. In the present study, we investigated the correlation between admission plasma VWF: Ag levels and long-term outcomes in patients with AMI.

## Materials and methods

### Study population

This was a single-center, prospective cohort study devised to assess whether admission VWF: Ag levels could prognosticated long-term outcomes in patients with AMI. The study complied with the Declaration of Helsinki, and the study protocol was approved by the Human Ethical Committee of the People's Hospital of Xinjiang Uygur Autonomous Region. All participants in this study provided written informed consent.

This study included 374 patients who were hospitalized due to chest pain and other discomfort and showed successfully improved coronary angiography after hospitalization (April 2018–December 2018). According to the inclusion criteria of the AMI and control groups, 209 patients were included in the AMI group and 165 patients were included in the control group. The diagnosis of AMI required the following criteria: symptoms consistent with AMI, elevated cardiac enzymes, including troponin T, troponin I, and/or creatinine kinase (at least a two-fold increase from the normal upper limit), and ST-segment elevation or depression in electrocardiograms compatible with AMI (17). Diagnostic ST elevation was defined as new ST elevation at the J point in at least two contiguous leads of 2 mm (0.2 mV), and AMI patients with ST elevation were diagnosed with STEMI (18). The exclusion criteria: at the same time, complicated with severe infection, hemopathy, malignant tumors, autoimmune diseases and other thrombotic diseases; a previous history of coagulation dysfunction; recent use of hormones or other drugs that affect platelet function; recent major foreign surgery or a large number of injuries; a large amount of gastrointestinal bleeding; and a previous history of pernicious anemia.

### Data collection

Data on age, sex, body mass index (BMI: kg/m^2^), hypertension history, diabetes history, smoking history, and drinking history were collected by an electronic pathology system. The peak values of cardiac enzymes such as calcium protein T, I, and CK-MB and BNP levels during hospitalization were collected; D-dimer, fibrinogen (FIB), platelet count (PLT), serum uric acid, serum creatinine, low-density lipoprotein cholesterol (LDL), high-density lipoprotein cholesterol (HDL), total cholesterol (TC), triglycerides (TGs), fasting blood glucose, and glycosylated hemoglobin (HbA1c) were also measured. Initial admission values such as left ventricular ejection fraction (LVEF), left ventricular diastolic volume, and systolic and diastolic blood pressure were recorded. The above hematological indices and super birth were uniformly tested by a standard method in the testing center and ultrasound department of the People's Hospital of Xinjiang Uygur Autonomous Region. This study was approved by the ethics committee of our hospital; participants were informed of the test items, provided informed consent, signed the informed consent form, and volunteered.

### Blood sample collection

Fasting peripheral venous blood samples was collected within 24 h after admission in the AMI group and within 24 h after coronary angiography in the control group. All blood samples were collected in 3-ml sodium citrate anticoagulant tubes and centrifuged at 3,500 r/min for 10 min immediately after collection. The upper plasma was separated and frozen into a tube and stored in a refrigerator at −80°C for centralized inspection. Subsequently, the plasma VWF: Ag levels of the patients were measured, in duplicate, using Quantikine Human Von Willebrand Factor (VWF) ELISA kits (CUSABIO Company, Wuhan, China) according to manufacturer's specifications at Xinjiang Key Laboratory of Cardiovascular Disease Research.

### Follow-up and outcomes

Patients with AMI were followed up by telephone from the day of admission. The follow-up in this study was terminated if major adverse cardiovascular events occurred, and the other patients were followed up for 2 years. During the telephone follow-up, the patients were asked about MACEs, including angina pectoris (with new ischaemic electrocardiographic ST-T changes), reinfarction (coronary angiography-confirmed in-stent acute and subacute thrombosis occlusion), acute heart failure (HF), and cardiac death (defined as death due to cardiac events such as malignant arrhythmia, cardiogenic shock and acute heart failure). Acute heart failure should be diagnosed by evidence of typical signs and symptoms of HF (dyspnoea, oedema, rales, third heart sound, jugular turgor, and lung congestion on chest X-rays). In patients who had more than one event, only the first event was counted. Deaths resulting from any event were counted.

### Statistical analyses

Continuous variables, if normally distributed, are presented as the means ± standard deviations ($\bar{x}\;\pm$ *s*) and were analyzed using the *T*-test; otherwise, they are presented as the median and 25th−75th percentiles and were analyzed by the Mann–Whitney *U*-test. Categorical variables are expressed as proportions (%) and frequencies (*n*) and were analyzed using the chi-square test. A receiver operating characteristic curve (ROC) was used to calculate the sensitivity, specificity and optimal cut-off value of plasma VWF: Ag for predicting MACEs in patients with acute myocardial infarction. According to the best cut-off value of plasma VWF: Ag, the patients were divided into two groups. The impact on survival was expressed by a Kaplan–Meier curve, and survival curves were compared by the log rank test. The variables that may affect prognosis were first analyzed by univariate Cox regression, and the meaningful variables in the univariate analysis were further analyzed by multivariate Cox regression. Differences were considered statistically significant at *P* < 0.05. The confidence interval was 95%, and the error range was 5%.

## Results

### Characteristics of AMI and control patients

An overview of the baseline demographics and clinical characteristics of the AMI and control groups can be found in Table 1. Table 1 shows that 374 patients underwent coronary angiography in the present study; among them, 209 were in the AMI group, and 165 were in the control group. Of the total population, 64.97% of the patients were men, and the mean age of the participants was 58.76 ± 13.21 years. Patients in the AMI group were an average of more than 6 years older than those in the control group (61.31 ± 12.20 vs. 55.20 ± 13.66 years). As far as BMI, hypertension history, SBP, DBP, and TG levels were concerned, the two groups had similar characteristics (all *P* > 0.05).

**Table 1:** Basic data comparison between patients in the AMI group and the control group.

**Variable**	**Total**	**AMI group**	**Control group**	***t*/χ^2^**	***P-*value**
	**(*n* = 374)**	**(*n* = 209)**	**(*n* = 165)**		
Age (years)	58.76 ± 13.21	61.37 ± 12.20	55.20 ± 13.66	4.608	**< 0.001**
Sex (male)	243 (64.97)	160 (76.60)	83 (50.30)	27.922	**< 0.001**
BMI (kg/m^2^)	26.00 (23.60, 28.10)	25.70 (23.70, 28.05)	26.00 (23.00, 29.00)	−0.601	0.548
Hypertension history	179 (49.18)	108 (51.67)	71 (43.03)	2.761	0.097
Diabetes	95 (25.40)	80 (38.28)	15 (9.09)	41.452	**< 0.001**
Smoking history	138 (36.89)	100 (47.85)	38 (23.03)	24.389	**< 0.001**
Drinking history	72 (19.25)	48 (22.97)	24 (14.55)	4.206	**0.040**
SBP (mmHg)	130.00 (115.00, 142.25)	128.00 (111.00, 143.50)	130.00 (118.50, 141.50)	−1.024	0.405
DBP (mmHg)	78.66 ± 13.98	77.63 ± 14.34	79.96 ± 13.46	−1.613	0.106
LDL (mmol/L)	2.66 ± 0.99	2.79 ± 1.05	2.49 ± 0.89	2.986	**0.003**
HDL (mmol/L)	1.03 ± 0.27	0.98 ± 0.25	1.09 ± 0.28	−4.247	**< 0.001**
TC (mmol/L)	4.21 ± 1.25	4.34 ± 1.34	4.04 ± 1.09	2.331	**0.020**
TG (mmol/L)	1.50 ± 1.14	1.54 ± 1.20	1.45 ± 1.05	0.728	0.467
LVEF (%)	54.48 ± 8.71	50.89 ± 7.99	59.04 ± 7.36	−10.138	**< 0.001**
BNP (pg/ml)	81.60 (11.98, 421.25)	224.00 (74.50, 743.50)	12.00 (5.00, 70.00)	6.465	**< 0.001**
FPG (mmol/L)	5.40 (4.70, 7.43)	6.80 (5.40, 9.70)	4.80 (4.40, 5.30)	10.229	**< 0.001**
HbA1c (%)	6.00 (5.70, 7.00)	6.10 (5.70, 7.6)	6.00 (6.00, 6.00)	5.588	**< 0.001**
VWF: Ag (IU/ml)	0.715 ± 0.331	0.860 ± 0.309	0.529 ± 0.258	11.273	**< 0.001**

HbA1c, glycosylated hemoglobin; LDL, low-density lipoprotein; HDL, high-density lipoprotein; TC, total cholesterol; TG, triglyceride; LVEF, left ventricular ejection fraction; BNP, brain natriuretic peptide; SBP, systolic blood pressure; DBP, diastolic blood pressure; FPG, fasting blood glucose.

Bold means that the p-value is less than 0.05 and is statistically significant.

### VWF: Ag levels are elevated in patients with AMI

**Figure 1:**
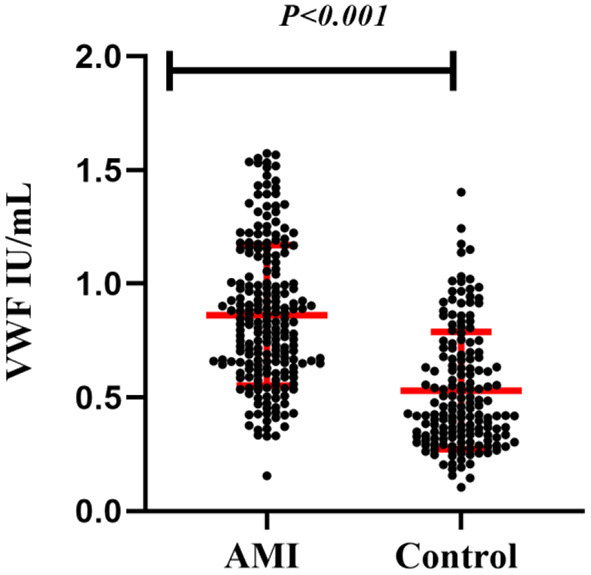
Comparison of plasma VWF: Ag levels ($\bar{x}\;\pm$ *s*) between patients with AMI and controls.

The mean VWF: Ag levels were nearly 1.63-fold higher in patients with AMI than in controls (0.860 ± 0.309 vs. 0.529 ± 0.258 IU/ml). On univariate analysis, this difference in the mean levels between the AMI and control groups for VWF (*P* < 0.001) was statistically significant (Figure 1).

### Characteristics of participants with and without MACEs

The baseline demographic and clinical characteristics of the MACE and non-MACE groups are summarized in Table 2. The two groups were similar in terms of drinking habits, hypertension, DM, and previous medication (aspirin, ADP receptor antagonists, ACEFs/ARBs, CCBs, statin treatment, and beta-blockers; all *P* > 0.05). Compared to those in the non-MACE group, patients in the MACE group were older (66.27 ± 11.79 vs. 58.37 ± 11.54 years, *P* < 0.001, Table 2), and their frequency of smoking habits was significantly higher (62.82 vs. 46.15%, *P* = 0.026, Table 2). In addition, more men were in the non-MACE group (67.95 vs. 82.69%, *P* = 0.020, Table 2).

**Table 2:** Comparison of basic clinical data between the MACE and non-MACE groups.

**Variable**	**MACE group**	**Non-MACE group**	***t*/χ^2^**	***P-*value**
	**(*n* = 78)**	**(*n* = 104)**		
Age (years)	66.27 ± 11.79	58.37 ± 11.54	4.530	**< 0.001**
Sex (male)	53 (67.95)	86 (82.69)	5.369	**0.020**
BMI (kg/m^2^)	25.37 ± 3.50	26.42 ± 3.35	−2.051	**0.042**
Hypertension history	40 (51.28)	49 (47.12)	0.310	0.578
Diabetes	44 (56.41)	69 (66.35)	1.869	1.172
Smoking history	49 (62.82)	48 (46.15)	4.974	**0.026**
Drinking history	65 (83.33)	75 (72.12)	3.160	0.075
Medication at home
Aspirin	74 (94.87)	101 (97.12)	0.607	0.436
ADP receptor antagonist	76 (97.44)	103 (99.04)	0.706	0.401
Beta blocker	71 (91.02)	90 (86.54)	0.879	0.348
ACEI/ARB	54 (69.23)	82 (78.85)	2.182	0.140
CCB	12 (15.39)	11 (10.58)	0.933	0.334
Statin treatment	76 (97.44)	98 (94.23)	2.696	0.101
STEMI	47 (60.26)	77 (74.04)	3.879	**0.048**
NSTEMI	31 (39.74)	27 (23.08)		
Killip class ≥3	37 (47.44)	42 (40.38)	0.902	0.342
Killip class ≤ 2	41 (52.56)	62 (59.62)		

Bold means that the p-value is less than 0.05 and is statistically significant.

Table 3 provides a summary of the laboratory parameters for the two patient groups. Neither group showed significant differences in CTnT, CK-MB, FIB, platelet count, Scr, or LVEF (all *P* > 0.05). The CTnI, D-dimer, uric acid, HbA1c, BNP, and Gensini scores were significantly higher in the MACE group than in the non-MACE group (*P* < 0.05, Table 3).

**Table 3:** Comparison of laboratory parameters between the MACE group and the non-MACE group.

**Variable**	**MACE group**	**Non-MACE group**	***t*/*Z***	***P-*value**
	**(*n* = 78)**	**(*n* = 104)**		
CTnT (ng/ml)	3.22 ± 3.01	5.68 ± 19.66	−1.092	0.276
CTnI (ng/ml)	5.96 (0.77, 16.03)	7.12 (0.83, 47.54)	−2.025	**0.045**
CK-MB (ng/ml)	22.55 (4.21, 59.50)	23.20 (4.45, 125.00)	−1.615	0.108
D-dimer (mg/L)	2.39 ± 3.67	1.11 ± 2.82	2.65	**0.009**
FIB (g/L)	4.04 ± 1.46	3.69 ± 1.31	1.707	0.090
Platelet count (10^9^/L)	220.44 ± 73.28	237.81 ± 77.74	−1.529	0.128
Uric acid (μmol/L)	362.00 (293.25, 464.00)	345.00 (288.00, 393.25)	2.838	**0.005**
Scr (μmol/L)	100.35 ± 74.60	101.92 ± 157.68	−0.081	0.936
HbA1c (%)	7.14 ± 1.88	6.59 ± 1.71	2.055	**0.041**
LVEF (%)	50.63 ± 7.36	50.92 ± 8.17	−0.251	0.802
BNP (pg/ml)	818.74 ± 1,110.37	508.29 ± 868.02	2.117	**0.036**
Gensini score	84.00 (64.75, 116.00)	56.50 (40.00, 72.00)	7.060	**< 0.001**
VWF: Ag (IU/ml)	1.088 ± 0.253	0.731 ± 0.252	9.441	**< 0.001**

Bold means that the p-value is less than 0.05 and is statistically significant.

### Elevated admission VWF: Ag levels predict clinical outcomes

**Figure 2:**
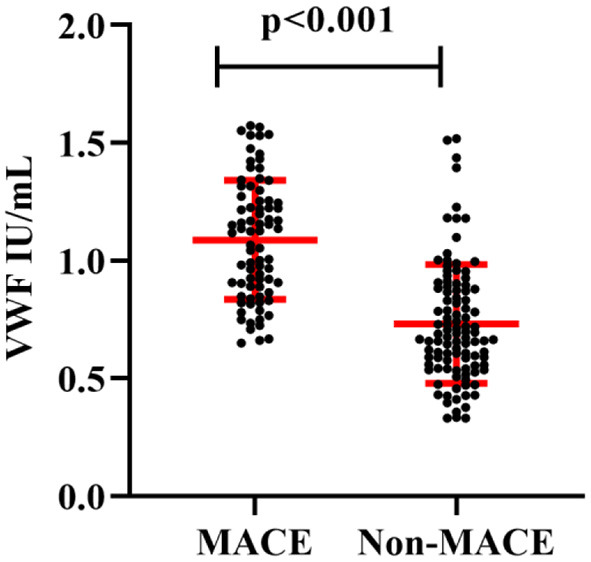
Comparison of plasma VWF: Ag levels ($\bar{x}\;\pm$ *s*) between the MACE and non-MACE groups.

An average of 19.3 months followed up was provided for 182 AMI patients after discharge from the hospital. There were 27 patients who lost contact during the follow-up period, and 78 patients (42.6%) developed MACEs, including cardiac death, MI but not fatal, and rehospitalization due to recurrent angina or heart failure. Patients with MACEs had higher admission plasma VWF: Ag levels than those without MACEs (1.088 ± 0.253 vs. 0.731 ± 0.252 IU/ml, *P* < 0.001; Figure 2).

**Figure 3:**
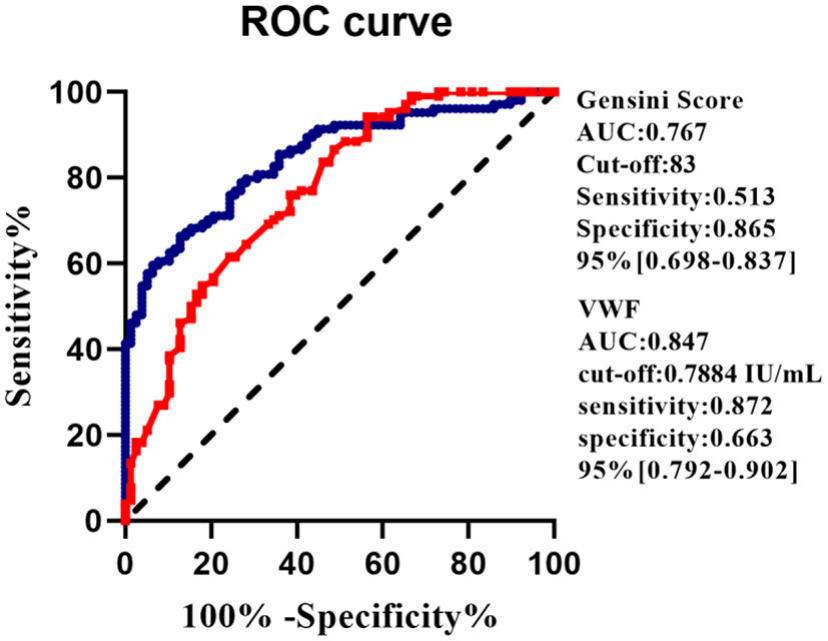
ROC curves of the VWF: Ag level and Gensini score for MACE prediction after 2 years of follow-up in patients with AMI. AUC, area under the curve; CI, confidence interval.

The receiver operating characteristics (ROC) of VWF: Ag for predicting MACEs in AMI patients are presented in Figure 3. It has an area under the ROC curve of 0.847 when predicting MACE. For predicting long-term MACEs, an optimal cut-off value for VWF: Ag was 0.7884 IU/ml, which had 87.2% sensitivity and 66.3% specificity. For comparison, a similar study examined the Gensini score and determined that it had a cut-off value of 83 for predicting the development of long-term MACEs with a sensitivity of 51.3% and a specificity of 86.5% [area under the curve (AUC): 0.767, 95%CI: 0.698–0.837, *P* = 0.001; Figure 3].

**Figure 4:**
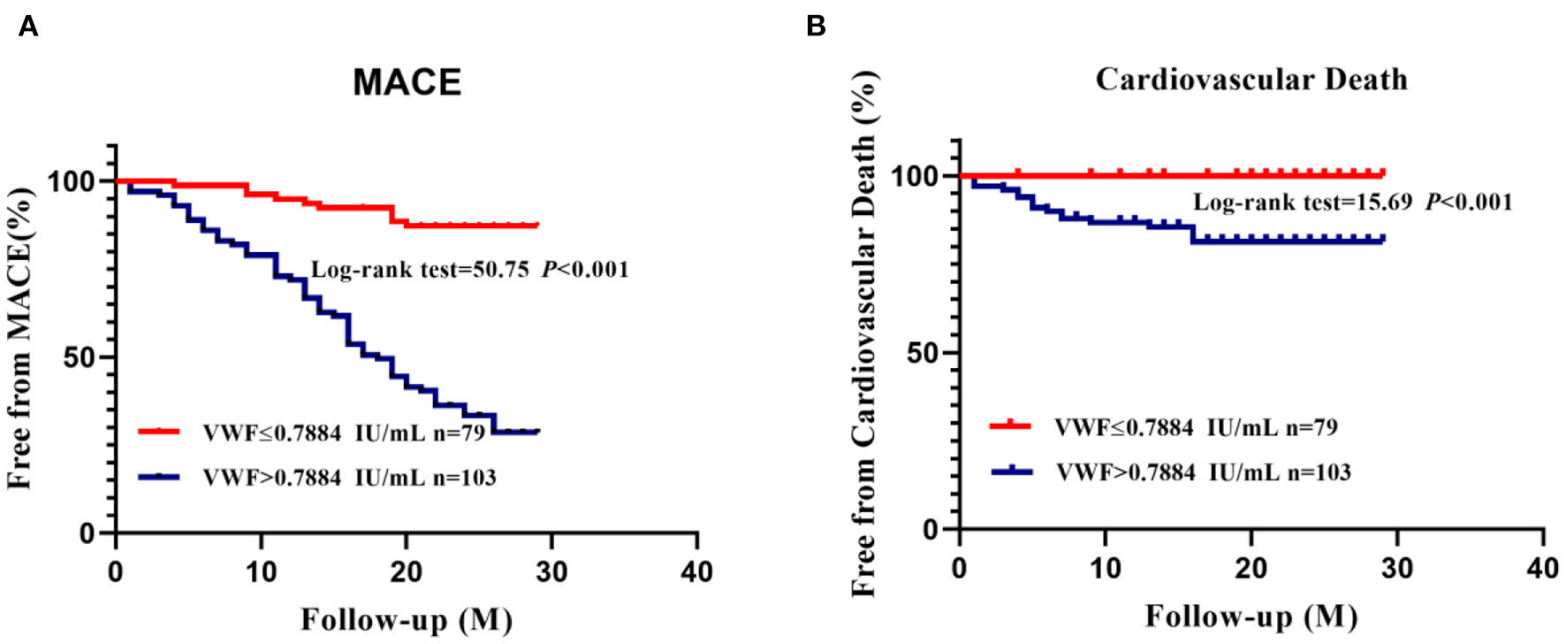
K–M curves for MACEs **(A)** and cardiovascular death **(B)** according to the best cut-off value of VWF: Ag.

Having reached the VWF: Ag cut-off value of 0.7884 IU/ml, patients discharged from the hospital were divided into two groups. Patients with high VWF: Ag levels were significantly more likely to experience MACEs than those with low VWF: Ag levels during the 2-year follow-up period, according to Kaplan-Meier curves (Figure 4A, *P* < 0.001).

As a result of Kaplan–Meier curves, it was evident that in the 2-year follow-up period, cardiovascular deaths were significantly more common among patients with high VWF: Ag levels than those with low VWF: Ag levels (Figure 4B, *P* < 0.001).

Univariate and multivariate analyses using stepwise Cox proportional hazards regression analysis (incorporating age, sex, BMI, smoking status, BNP, Gensini sore, HbA1c, D-dimer, and uric acid as putative predictors) confirmed that VWF: Ag levels remained an independent predictor of MACEs, especially in patients of VWF: Ag >0.0788, yielding a higher event risk (HR = 7.993, 95%CI: 4.095–15.603 and HR = 6.004, 95%CI: 2.987–12.070, *P* < 0.001, respectively; Table 4).

**Table 4:** Univariate and multivariate Cox regression analyses predicting the occurrence of MACEs in patients with AMI after 2 years of follow-up.

**Variable**	**Univariate analysis**		**Multivariate analysis**	
	**HR (95% CI)**	***P-*value**	**HR (95% CI)**	***P-*value**
VWF: Ag ≤ 0.0788	Ref	–	Ref	–
VWF: Ag > 0.0788	7.993 (4.095–15.603)	< 0.001	6.004 (2.987–12.070)	**< 0.001**
Gensini score	1.027 (1.019–1.034)	< 0.001	1.018 (1.010–1.026)	**< 0.001**
Age	1.043 (1.024–1.063)	< 0.001	1.023 (1.003–1.043)	**0.026**
BMI	0.922 (0.858–0.90)	0.026	0.984 (0.909–1.064)	0.685
Sex (men)	0.590 (0.367–0.951)	0.030	1.208 (0.667–2.189)	0.533
Smoking	0.625 (0.395–0.989)	0.045	1.048 (0.601–1.830)	0.868
BNP	1.001 (1.001–1.002)	0.014	1.00 (1.000–1.000)	0.513
HbA1c (%)	1.133 (1.013–1.266)	0.028	1.056 (0.930–1.199)	0.403
D-dimer (mg/L)	1.082 (1.033–1.133)	0.001	1.032 (0.953–1.117)	0.440
Uric acid (μmol/L)	1.003 (1.001–1.005)	0.001	1.002 (1.000–1.004)	0.058

CI, confidence interval; HR, hazard ratio; other abbreviations as in Table 1.

Bold means that the p-value is less than 0.05 and is statistically significant.

AMI patients with higher VWF: Ag levels tended to have a worse prognosis. During the 2 years of follow-up, 78 (42.6%) patients in the AMI group experienced MACEs, and more patients died from cardiovascular disease in the patient group with higher VWF: Ag levels. Furthermore, higher VWF: Ag levels were significantly associated with an increased risk for MACEs (*P* < 0.001; Figure 4A) and cardiovascular death (*P* < 0.001; Figure 4B) according to K–M curves and log-rank tests. Both univariate and multivariable Cox proportional hazard models further indicated the prognostic value of VWF: Ag as a continuous variable, with higher VWF: Ag levels being independently associated with the risk for MACEs. Moreover, when the VWF: Ag levels were divided into two groups according to a VWF: Ag cut-off value of 0.7884 IU/ml, a 6.004 fold increased risk of MACEs was noted (HR = 6.004 95%CI: 2.987–12.070) per SD, *P* < 0.01, Table 4) in the model fully adjusted for conventional risk factors for cardiovascular disease.

## Discussion

The endothelium modulates the interaction between the circulating blood, the vascular wall, and the surrounding tissue. VWF levels are thought to be a vital indicator for endothelial dysfunction, after release of VWF from storage, some endothelial derived VWF multimers remain anchored to the surface of endothelial cells, forming string-like structures, which, under normal flow, elongate the VWF multimers from a globular to a string-like form, thereby exposing the cleavage site in the A2 domain to the metalloproteinase ADAMTS13 (a disintegrin and metalloproteinase with a thrombospondin type 1 motif, member 13). Under conditions of high fluid shear stress, endothelial bound ULVWF strings are cleaved multiple times by ADAMTS13 to shorter multimers that are still ultra-large in size, suggesting that VWF undergoes further ADAMTS13-mediated proteolysis in circulation (10, 19–23). A crucial factor in haemostasis, thrombosis, and vascular inflammation is VWF, a large multimeric glycoprotein (24, 25).

The VWF test has been used primarily to diagnose von Willebrand disease (VWD), which is characterized by quantitative and/or qualitative defects in the VWF (26). These tests include the assessment of VWF antigen (VWF: Ag), VWF ristocetin cofactor activity assays, ristocetin-triggered platelet glycoprotein Ib binding assays, ristocetin-induced platelet agglutination assays, VWF-collagen binding assays (VWF:CB), and VWF-factor VIII binding assays. The current assays are mostly based on ELISA, while the new flow cytometry and chemiluminescence immunoassays are not yet available for clinical use. VWFlevels are influenced by blood type, whereas this limitation does not exist for pre-VWF peptides (27). AMI patients have not only significantly more VWF, but also significantly more ULVWF than normal, as well as increased activity. The increased level of ULVWF and the resulting stronger adhesion and aggregation may contribute to the aggressiveness of AMI. In the present study we measured plasma VWF: Ag levels. The association between VWF: Ag levels and coronary artery disease is well-established, suggesting that endothelial dysfunction may contribute to its pathogenesis. Patients with previous myocardial infarction, for instance, have higher VWF concentrations (28–30).

It was demonstrated in a prospective multicentre study involving 3,043 patients with stable angina or previous MI that higher plasma VWF levels were associated with an 8.5% higher rate of MI and sudden cardiac death (31). As part of this study, we explored whether plasma VWF levels could be used to predict long-term clinical outcomes in patients with AMI.

Findings of major importance were discovered. First, the plasma VWF: Ag levels in patients with AMI were elevated when compared with healthy controls, and those who died were found to have higher plasma VWF: Ag levels than those who survived. Second, patients with higher plasma VWF: Ag levels had a higher mortality rate as well as a higher likelihood of developing MACEs than those with lower VWF: Ag levels. Third, patients with higher plasma VWF: Ag levels experienced significantly more MACEs during the 2-year follow-up period. Thus, there is evidence that plasma VWF: Ag levels appear to be predictive of long-term outcome in patients with AMI.

In this study, 209 patients with AMI were followed up. During the 2-year follow-up period, 26 patients were lost to follow-up for various reasons. Of the 182 patients who were successfully followed up, 78 patients (42.6%) had MACEs, 20 of whom died due to various cardiac causes. Heart failure occurred in 19 patients during the follow-up. During the follow-up period, 27 patients had symptoms of discomfort, such as chest pain, chest tightness and shortness of breath, and were definitively diagnosed with angina pectoris in the hospital or outpatient department. Twelve patients developed myocardial infarction again during the follow-up and underwent revascularization.

The plasma VWF: Ag level in AMI patients in our study increased 1.63-fold relative to the levels in healthy participants. In a previous study, VWF: Ag plasma levels were investigated as potential biomarkers for AMI and may also be associated with recurrent MI (32). Another study suggested that the coronary plaque burden was associated with VWF: Ag levels in stable angina patients (SAP) undergoing coronary angiography (33). A correlation has been established between VWF: Ag and plaque instability or severity of coronary artery lesions. In patients who had a single stent implanted, VWF: Ag levels increased slightly following the procedure. VWF: Ag plasma levels rose steeply in patients with multiple-stent implantations, from 112.7 ± 25.16 to 152.78 ± 41.03 IU/dl. Thus, multiple stenting is dramatically raised the plasma levels of VWF: Ag in the coronary circulation (34). The Gensini score, a comprehensive measure for the severity of diseased arteries, showed, however, that higher VWF levels resulted in higher Gensini scores.

The presence of high VWF levels was predictive of adverse cardiovascular outcomes and death in ACS and SAP patients 1 year after diagnosis (33). In acute coronary syndrome (ACS), rapid rises in VWF are associated with poor outcome (35, 36). There is less research on plasma VWF: Ag levels' predictive value in patients with AMI, but our study showed that a higher plasma VWF: Ag level of >0.7884 IU/ml predicts long-term MACEs and cardiovascular death in AMI patients. As previously observed by a study which included 314 patients with STEMI, our results support the observation that VWF increases are associated with adverse cardiac events in STEMI patients and further demonstrating novel associations between symptom duration, coronary flow, and the subsequent increase in VWF (36).

The results of the current study were confirmed by comparing the MACE and non-MACE groups. In the MACE group, all traditional myocardial injury markers, Gensini scores, Scr, HbA1c and BNP were significantly higher than those in the non-MACE group. Overall, AMI patients who died after discharge from the hospital had markedly higher plasma VWF: Ag levels than survivors. Based on the results of the present study, plasma VWF: Ag continues to be an independent indicator of long-term adverse events in patients with AMI. The risk of adverse events in patients with high VWF: Ag levels was approximately 6.004-fold higher than that in patients with lower VWF: Ag.

Our study had some limitations. As a first step, this was an observational study conducted at one center with limited enrollment of AMI patients, and blood was sampled after coronary angiography. Neither the time interval between the occurrence of an AMI nor the timing of admission were strictly confined, and only the level of plasma VWF at admission was analyzed. As the disease progresses, VWF may fluctuate with different clinical outcomes. In regards to VWF, there are no follow-up data available. In addition, the detection of VWF mainly includes its antigen and activity, where VWF:GPIbR assay is to study the activity between VWF and platelet surface glycoprotein Ib, and VWF: CB is to study the activity between VWF and vascular endothelial matrix collagen (26). In future studies, we will combine VWF: Ab and VWF activity to provide a better theoretical basis and target for the prediction, development, prognosis, and endothelial function of AMI and acute thrombotic events. Subsequently, our study did not examine MRI to measure the size of infarcts, and other clinical characteristics, such as plaque vulnerability, should be taken into consideration in addition to GS when evaluating the severity of AMI. Instead, we applied only the well-recognized cardiac enzymatic biomarker as a surrogate to assess the degree of myocardial injury.

As a result of AMI, significantly higher plasma VWF: Ag levels are observed. During the 2-year follow-up, patients with higher plasma VWF: Ag levels experienced a higher risk of MACEs. Plasma VWF: Ag levels remained an independent predictor of long-term MACEs in AMI patients after accounting for traditional risk factors.

## Data availability statement

The original contributions presented in the study are included in the article/supplementary material, further inquiries can be directed to the corresponding author.

## Ethics statement

The studies involving human participants were reviewed and approved by People's Hospital of Xinjiang Uygur Autonomous Region. The patients/participants provided their written informed consent to participate in this study.

## Author contributions

There was substantial collaboration between all the authors on this paper, with ZX and HP designing the study. ZX, Y-XZ, S-WT, CK, DA, and GH contributed to the acquisition, analysis, and interpretation of data. ZX, Y-XZ, and HP were involved in developing and revising the manuscript. Upon completion of the final review and approval of the manuscript, all authors signed off on it.

## References

[1] Ma LY Chen WW Gao RL Liu LS Zhu ML Wang YJ. China cardiovascular diseases report 2018: an updated summary. J Geriatr Cardiol. (2020) 17:1–8. 10.11909/j.issn.1671-5411.2020.01.001 32133031 PMC7008101

[2] Hansson GK Hermansson A. The immune system in atherosclerosis. Nat Immunol. (2011) 12:204–12. 10.1038/ni.2001 21321594

[3] Rafieian-Kopaei M Setorki M Doudi M Baradaran A Nasri H. Atherosclerosis: process, indicators, risk factors and new hopes. Int J Prev Med. (2014) 5:927–46. 25489440 PMC4258672

[4] Nesbitt WS Westein E Tovar-Lopez FJ Tolouei E Mitchell A Fu J. A shear gradient-dependent platelet aggregation mechanism drives thrombus formation. Nat Med. (2009) 15:665–73. 10.1038/nm.1955 19465929

[5] Zhou Z Nguyen TC Guchhait P Dong JF. Von Willebrand factor, ADAMTS-13, and thrombotic thrombocytopenic purpura. Semin Thromb Hemost. (2010) 36:71–81. 10.1055/s-0030-1248726 20391298

[6] Lenting PJ Christophe OD Denis CV. von Willebrand factor biosynthesis, secretion, and clearance: connecting the far ends. Blood. (2015) 26:2019–28. 10.1182/blood-2014-06-528406 25712991

[7] Zheng XL. ADAMTS13 and von Willebrand factor in thrombotic thrombocytopenic purpura. Annu Rev Med. (2015) 66:211–25. 10.1146/annurev-med-061813-013241 25587650 PMC4599565

[8] Leebeek FW Eikenboom JC. Von Willebrand's disease. N Engl J Med. (2016) 375:2067–80. 10.1056/NEJMra1601561 27959741

[9] Nichols TC Bellinger DA Tate DA Reddick RL Read MS Koch GG. von Willebrand factor and occlusive arterial thrombosis. A study in normal and von Willebrand's disease pigs with diet-induced hypercholesterolemia and atherosclerosis. Arteriosclerosis. (1990) 10:449–61. 10.1161/01.ATV.10.3.449 2344301

[10] Fredrickson BJ Dong JF McIntire LV López JA. Shear-dependent rolling on von Willebrand factor of mammalian cells expressing the platelet glycoprotein Ib-IX-V complex. Blood. (1998) 92:3684–93. 10.1182/blood.V92.10.3684 9808562

[11] Lip GY Blann A. von Willebrand factor: a marker of endothelial dysfunction in vascular disorders? Cardiovasc Res. (1997) 34:255–65. 10.1016/S0008-6363(97)00039-4 9205537

[12] Wang X Zhao J Zhang Y Xue X Yin J Liao L. Kinetics of plasma von Willebrand factor in acute myocardial infarction patients: a meta-analysis. Oncotarget. (2017) 8:90371–9. 10.18632/oncotarget.20091 29163836 PMC5685757

[13] Li Y Li L Dong F Guo L Hou Y Hu H. Plasma von Willebrand factor level is transiently elevated in a rat model of acute myocardial infarction. Exp Ther Med. (2015) 10:1743–9. 10.3892/etm.2015.2721 26640545 PMC4665708

[14] Sakai H Goto S Kim JY Aoki N Abe S Ichikawa N. Plasma concentration of von Willebrand factor in acute myocardial infarction. Thromb Haemost. (2000) 84:204–9. 10.1055/s-0037-1613997 10959690

[15] Kovacevic KD Mayer FJ Jilma B Buchtele N Obermayer G Binder CJ. Von Willebrand factor antigen levels predict major adverse cardiovascular events in patients with carotid stenosis of the ICARAS study. Atherosclerosis. (2019) 290:31–6. 10.1016/j.atherosclerosis.2019.09.003 31557676

[16] Fan M Wang X Peng X Feng S Zhao J Liao L. Prognostic value of plasma von Willebrand factor levels in major adverse cardiovascular events: a systematic review and meta-analysis. BMC Cardiovasc Disord. (2020) 10:72. 10.1186/s12872-020-01375-7 32039706 PMC7011353

[17] Watanabe Y Sakakura K Taniguchi Y Yamamoto K Wada H Momomura SI. Determinants of slow flow in percutaneous coronary intervention to the culprit lesion of non-ST elevation myocardial infarction. Int Heart J. (2018) 59:1237–45. 10.1536/ihj.18-050 30305588

[18] Tsukui T Sakakura K Taniguchi Y Yamamoto K Wada H Momomura SI. Determinants of short and long door-to-balloon time in current primary percutaneous coronary interventions. Heart Vessels. (2018) 33:498–506. 10.1007/s00380-017-1089-x 29159569

[19] Li M Goto S Sakai H Kim JY Ichikawa N Yoshida M. Enhanced shear-induced von Willebrand factor binding to platelets in acute myocardial infarction. Thromb Res. (2000) 100:251–61. 10.1016/S0049-3848(00)00326-1 11113268

[20] Reininger AJ. Function of von Willebrand factor in haemostasis and thrombosis. Haemophilia. (2008) 14(Suppl 5):11–26. 10.1111/j.1365-2516.2008.01848.x 18786007

[21] Paulinska P Spiel A Jilma B. Role of von Willebrand factor in vascular disease. Hamostaseologie. (2009) 29:32–8. 10.1055/s-0037-1616936 19151843

[22] Levy GG Nichols WC Lian EC Foroud T McClintick JN McGee BM. Mutations in a member of the ADAMTS gene family cause thrombotic thrombocytopenic purpura. Nature. (2001) 4:488–94. 10.1038/35097008 11586351

[23] De Ceunynck K Rocha S Feys HB De Meyer SF Uji-i H Deckmyn H. Local elongation of endothelial cell-anchored von Willebrand factor strings precedes ADAMTS13 protein-mediated proteolysis. J Biol Chem. (2011) 21:36361–7. 10.1074/jbc.M111.271890 21896483 PMC3196129

[24] Franchini M Mannucci PM. Von Willebrand factor: another janus-faced hemostasis protein. Semin Thromb Hemost. (2008) 34:663–9. 10.1055/s-0028-1104545 19085767

[25] Lenting PJ Casari C Christophe OD Denis CV. von Willebrand factor: the old, the new and the unknown. J Thromb Haemost. (2012) 10:2428–37. 10.1111/jth.12008 23020315

[26] Favaloro EJ Pasalic L Curnow J. Laboratory tests used to help diagnose von Willebrand disease: an update. Pathology. (2016) 48:303–18. 10.1016/j.pathol.2016.03.001 27131932

[27] Marianor M Zaidah AW. Maraina ChC. von Willebrand factor propeptide: a potential disease biomarker not affected by ABO blood groups. Biomark Insights. (2015) 23:75–9. 10.4137/BMI.S24353 26339184 PMC4548735

[28] Hamsten A Blombäck M Wiman B Svensson J Szamosi A de Faire U. Haemostatic function in myocardial infarction. Br Heart J. (1986) 55:58–66. 10.1136/hrt.55.1.58 3947483 PMC1232069

[29] Haines AP Howarth D North WR Goldenberg E Stirling Y Meade TW. Haemostatic variables and the outcome of myocardial infarction. Thromb Haemost. (1983) 50:800–3. 10.1055/s-0038-1665316 6198743

[30] Lip GY Lowe GD Metcalfe MJ Rumley A Dunn FG. Effects of warfarin therapy on plasma fibrinogen, von Willebrand factor, and fibrin D-dimer in left ventricular dysfunction secondary to coronary artery disease with and without aneurysms. Am J Cardiol. (1995) 76:453–8. 10.1016/S0002-9149(99)80129-5 7653443

[31] Thompson SG Kienast J Pyke SD Haverkate F van de Loo JC. Hemostatic factors and the risk of myocardial infarction or sudden death in patients with angina pectoris. European Concerted Action on Thrombosis and Disabilities Angina Pectoris Study Group. N Engl J Med. (1995) 332:635–41. 10.1056/NEJM199503093321003 7845427

[32] Rutten B Maseri A Cianflone D Laricchia A Cristell NA Durante A. Plasma levels of active Von Willebrand factor are increased in patients with first ST-segment elevation myocardial infarction: a multicenter and multiethnic study. Eur Heart J Acute Cardiovasc Care. (2015) 4:64–74. 10.1177/2048872614534388 24833640

[33] Sonneveld MA Cheng JM Oemrawsingh RM de Maat MP Kardys I Garcia-Garcia HM. Von Willebrand factor in relation to coronary plaque characteristics and cardiovascular outcome. Results of the ATHEROREMO-IVUS study. Thromb Haemost. (2015) 113:577–84. 10.1160/TH14-07-0589 25472874

[34] Heper G Murat SN Durmaz T Kalkan F. Prospective evaluation of von Willebrand factor release after multiple and single stenting. Angiology. (2004) 55:177–86. 10.1177/000331970405500210 15026873

[35] Lee KW Lip GY Tayebjee M Foster W Blann AD. Circulating endothelial cells, von Willebrand factor, interleukin-6, and prognosis in patients with acute coronary syndromes. Blood. (2005) 105:526–32. 10.1182/blood-2004-03-1106 15374879

[36] Collet JP Montalescot G Vicaut E Ankri A Walylo F Lesty C. Acute release of plasminogen activator inhibitor-1 in ST-segment elevation myocardial infarction predicts mortality. Circulation. (2003) 108:391–4. 10.1161/01.CIR.0000083471.33820.3C 12860898

